# Characterisation of AmphiAmR11, an Amphioxus (*Branchiostoma floridae*) D_2_-Dopamine-Like G Protein-Coupled Receptor

**DOI:** 10.1371/journal.pone.0080833

**Published:** 2013-11-12

**Authors:** Asha L. Bayliss, Peter D. Evans

**Affiliations:** The Signalling Laboratory, the Babraham Institute, Cambridge, United Kingdom; Goethe University Frankfurt, Germany

## Abstract

The evolution of the biogenic amine signalling system in vertebrates is unclear. However, insights can be obtained from studying the structures and signalling properties of biogenic amine receptors from the protochordate, amphioxus, which is an invertebrate species that exists at the base of the chordate lineage. Here we describe the signalling properties of AmphiAmR11, an amphioxus (*Branchiostoma floridae*) G protein-coupled receptor which has structural similarities to vertebrate α_2_-adrenergic receptors but which functionally acts as a D_2_ dopamine-like receptor when expressed in Chinese hamster ovary -K1 cells. AmphiAmR11 inhibits forskolin-stimulated cyclic AMP levels with tyramine, phenylethylamine and dopamine being the most potent agonists. AmphiAmR11 also increases mitogen-activated protein kinase activity and calcium mobilisation, and in both pathways, dopamine was found to be more potent than tyramine. Thus, differences in the relative effectiveness of various agonists in the different second messenger assay systems suggest that the receptor displays agonist-specific coupling (biased agonism) whereby different agonists stabilize different conformations of the receptor which lead to the enhancement of one signalling pathway over another. The present study provides insights into the evolution of α_2_-adrenergic receptor signalling and support the hypothesis that α_2_-adrenergic receptors evolved from D_2_-dopamine receptors. The AmphiAmR11 receptor may represent a transition state between D_2_-dopamine receptors and α_2_-adrenergic receptors.

## Introduction

Studying the complement of biogenic amine receptors in the cephalochordate, amphioxus, can provide insight into the evolution of the biogenic amine signalling system present in vertebrates. Amphioxus is an invertebrate species that exists at the base of the chordate lineage [1-3] and is considered to be the ‘missing link’ between invertebrates and vertebrates [4]. It is predicted to have 44 biogenic amine receptors [5,6] and expresses high levels of octopamine, dopamine and serotonin, and trace levels of noradrenaline [7]. The characterisation of three of the 44 predicted biogenic amine receptors, AmphiD_1_/β, AmphiAmR1 and AmphiAmR2, showed that amphioxus has both vertebrate-type and invertebrate-type D_1_-dopamine receptors [8,9]. Of interest to the current study is the identification of five amphioxus receptors, AmphiAmR4-6, -9 and -11, which cluster off the main branch of a phylogenetic tree leading to human and mouse α_2_-adrenergic receptors (α_2_-ARs) [5]. The characterisation of one of the five receptors, AmphiAmR4, showed that amphioxus has at least one α_2_-adrenregic-like receptor and implied that an adrenergic signalling system, albeit a simple one, likely exists in amphioxus [10]. 

Predicting a receptor’s function based on sequence similarity to known receptor types can provide useful information on the potential endogenous ligand of the receptor or the pathways it is likely to couple to. However, these predictions have not always been accurate in detailing a receptor’s function. For example, DmDopEcR from Drosophila and AmphiD_1_/β from amphioxus showed highest sequence similarity to vertebrate β-adrenergic receptors but upon their characterisation, both were found to be most similar to D_1_-dopamine receptors [9,11]. In addition, the amphioxus receptor, AmphiAmR4, was shown to have highest sequence similarity to octopamine/tyramine receptors in BLAST and HMM analyses [5], but was found to be functionally more similar to α_2_-adrenergic receptors upon its characterisation [10]. Thus, it is important to fully characterise a receptor to gain insight into its function.

One reason why it is difficult to predict a receptor’s function based on sequence similarity is that the biogenic amine receptors share a relatively high level of sequence similarity. For example, D_1_- and D_2_-dopamine receptors have higher sequence similarity to β- and α_2_-ARs, respectively, than to each other [12-14]. In fact, the α_2_-ARs are thought to have evolved from D_2_-like receptors [12,13] and hence, the two receptor classes have some similar functional properties. Both receptor classes couple to pertussis toxin-sensitive G_i_ proteins and, thus, primarily couple to a decrease in intracellular cAMP levels. In addition, α_2_-adrenergic and D_2_-dopamine receptors have both been shown to mediate ERK1/2 activation, an increase in intracellular calcium levels, opening of potassium channels and closing of voltage-dependent calcium channels, amongst other pathways [15-18]. 

The present study describes the pharmacological characterisation of the amphioxus receptor, AmphiAmR11. BLAST and phylogenetic analyses show that AmphiAmR11 has highest sequence similarity to α_2_-ARs [5]. However, HMM analysis suggests the receptor sequence is most similar to 5HT_1A_, Oct/Tyr or D_2_-dopamine receptors [5]. Thus, it is of interest to determine which family of biogenic amine receptors AmphiAmR11 is most similar to in order to provide more insight into the evolution of the vertebrate biogenic amine receptors. AmphiAmR11 was expressed in Chinese hamster ovary (CHO)-K1 cells and screened for its ability to regulate adenylyl cyclase activity, to mediate mitogen activated protein kinase (MAPK) activation and to induce calcium mobilisation in response to stimulation by various biogenic amines, synthetic agonists and synthetic antagonists. 

## Materials and Methods

### Cloning of AmphiAmR11

AmphiAmR11 was initially amplified from adult amphioxus (*Branchiostoma floridae*) CNS, head and muscle cDNA libraries using the Advantage 2 PCR system (BD Biosciences, Oxford, UK) [19]. The libraries were kindly supplied by Dr M. Matz, Whitney Laboratory University of Florida, Saint Augustine, Florida, USA, (current address Integrative Biology, University of Texas at Austin, Texas, USA). The AmphiAmR11 open reading frame (ORF) was amplified from a pcDNA3.1/CT-GFP vector (supplied by Dr C. Burman) using PCR and subcloned into the expression vector pcDNA3.1/V5-His (Invitrogen Life Sciences, Paisley, UK) using TA cloning. The forward primer (5’-gctacaatgggtgtttttgc-3’) was based on the 5’ ORF containing the start codon and the reverse primer (5’-atggctctgacatttacaac-3’) was based on the 3’ORF excluding the stop codon.

### Accession number

The EMBL database accession number for AmphiAmR11 is AM396599. 

### Expression in mammalian cell lines

CHO-K1 cells were maintained in Ham’s F-12 nutrient media (Invitrogen Life Sciences) supplemented with 10% charcoal-stripped foetal bovine serum (CS-FBS) (Hyclone, Cramlington, UK), 50 U/μg penicillin/streptomycin and 4 mM L-glutamine (Invitrogen Life Sciences) in 5% CO_2_ at 37°C. Stably transfected cell lines were generated by the transfection of CHO-K1 cells using Lipofectamine (Invitrogen Life Sciences) as described previously [8,9]. Immunocytochemistry was performed with an anti-V5-FITC antibody as described previously (20) to confirm the expression of AmphiAmR11 in the CHO-K1 cell lines and to identify the receptor’s localisation within the cells. The AmphiAmR11 receptor showed high expression at the cell periphery which was essential for its function as a biogenic amine receptor since the biogenic amines are believed to be cell impermeable.

### cAMP determination

cAMP levels in stably transfected CHO-K1 cells were determined as described previously [8,9,21], except 100 µM isobutylmethylxanthine (IBMX) was used. cAMP levels are represented as a percentage of basal samples unless otherwise stated. Student’s T-test (two-tailed and unpaired) was used to test for significance. Unless otherwise stated, all data are shown as mean ± SEM. Each data point plotted was the mean of data obtained from at least three experiments. Within each experiment three separate replicate wells were analysed for each condition and the cAMP assays on each of the wells was carried out in duplicate. 

Forskolin was used both to increase basal cAMP levels to make it easier to detect increases and decreases in cAMP levels in the same experiments and also to potentiate responses to agonists to more accurately determine their threshold effects [see 22]. A non-saturating 10 µM concentration of forskolin was used. Basal levels of CHO cell cyclic AMP were 5.1 ± 1.7 pmoles / mg protein (n = 3) and these were raised to 1053.7 ± 4.8 pmoles / mg protein after exposure to 10 µM forskolin. Protein levels were determined using a Bradford assay.

### Phospho-ERK determination

Phospho-extracellular signal-related kinase (Phospho-ERK) levels were determined essentially as described previously [11,23,24]. Minor modifications have been described [8,9]. ERK1/2 phosphorylation levels were quantified by densitometry. The developed films were scanned in and analysed using the software program, Aida. The data generated by Aida was then processed using Microsoft Excel. Values for the kinase expression and activity levels were defined as 100% in the control samples. Student’s T-test (two-tailed and unpaired) was used to test for significance. 

### Intracellular calcium measurements

Stably transfected CHO-K1 cells were assayed for changes in intracellular calcium levels using an Olympus Cell^R imaging system and the fluorescent indicator Fura-2, as described previously [25], with modifications described by Bayliss et al. [20]. At 1 minute into the experiment, a buffer control was added and at 2 minutes into the experiment, the specified concentration of agonist was added. A single coverslip of cells was used per concentration of agonist and the percentage of cells that responded per field of view was calculated. The results from each coverslip gave an *n* of 1 and each agonist concentration was repeated at least three times on different days. 

### Drugs

The drugs used in these experiments were obtained from the following sources: dopamine hydrochloride, (**-**)-noradrenaline hydrochloride, (**-**)-adrenaline, tyramine hydrochloride, (±)-*p*-octopamine hydrochloride, (±)-synephrine, (±)-1-phenylethylamine, 5-HT, histamine dihydrochloride, IBMX, (+)-butaclamol hydrochloride, (±)-6-chloro-APB [(±)-6-chloro-7,8-dihydroxy-3-allyl-1-phenyl-2,3,4,5-tetrahydro-^1^H-3-benzazepine hydrobromide], clonidine hydrochloride, (±)-isoproterenol hydrochloride, naphazoline hydrochloride, metoclopramide hydrochloride, mianserin hydrochloride, pertussis toxin, phentolamine hydrochloride, (R)-(-)-phenylephrine hydrochloride, prazosin hydrochloride, chlorpromazine, (-)-quinpirole hydrochloride, rauwolscine hydrochloride, *R*(+)-SKF-38393 [*R*(+)-1-phenyl-2,3,4,5-tetrahydro-(^1^H)-3-benzazepine-7,8-diol], *R*(+)SCH-23390 [*R*(+)-7-chloro-8-hydroxy-3-methyl-1-phenyl-2,3,4,5-tetrahydro-^1^H-3-benzazepine hydrochloride], spiperone hydrochloride, tyrphostin AG1296, U0126 [1,4-Diamino-2,3-dicyano-1,4-bis(o-aminophenylmercapto)butadiene], UK14304, WB4101 and yohimbine hydrochloride were from Sigma-Aldrich (Poole, Dorset, UK); *cis*-(*Z*)-flupenthixol dihydrochloride was from Research Biochemicals (Natick, MA). Forskolin was obtained from Abcam Biochemicals (Cambridge, UK). LY294002, tyrphostin AG 1478, Ro 31-8220, PP2 [3-(4-chlorophenyl) 1-(1,1-dimethylethyl)-1*H*-pyrazolo[3,4-*d*]pyrimidin-4-amine] and Fura 2-AM were purchased from Tocris Bioscience (Bristol, UK). 

## Results

### Coupling of AmphiAmR11 to adenylyl cyclase activity

#### Biogenic amine specificity

**Figure 1:**
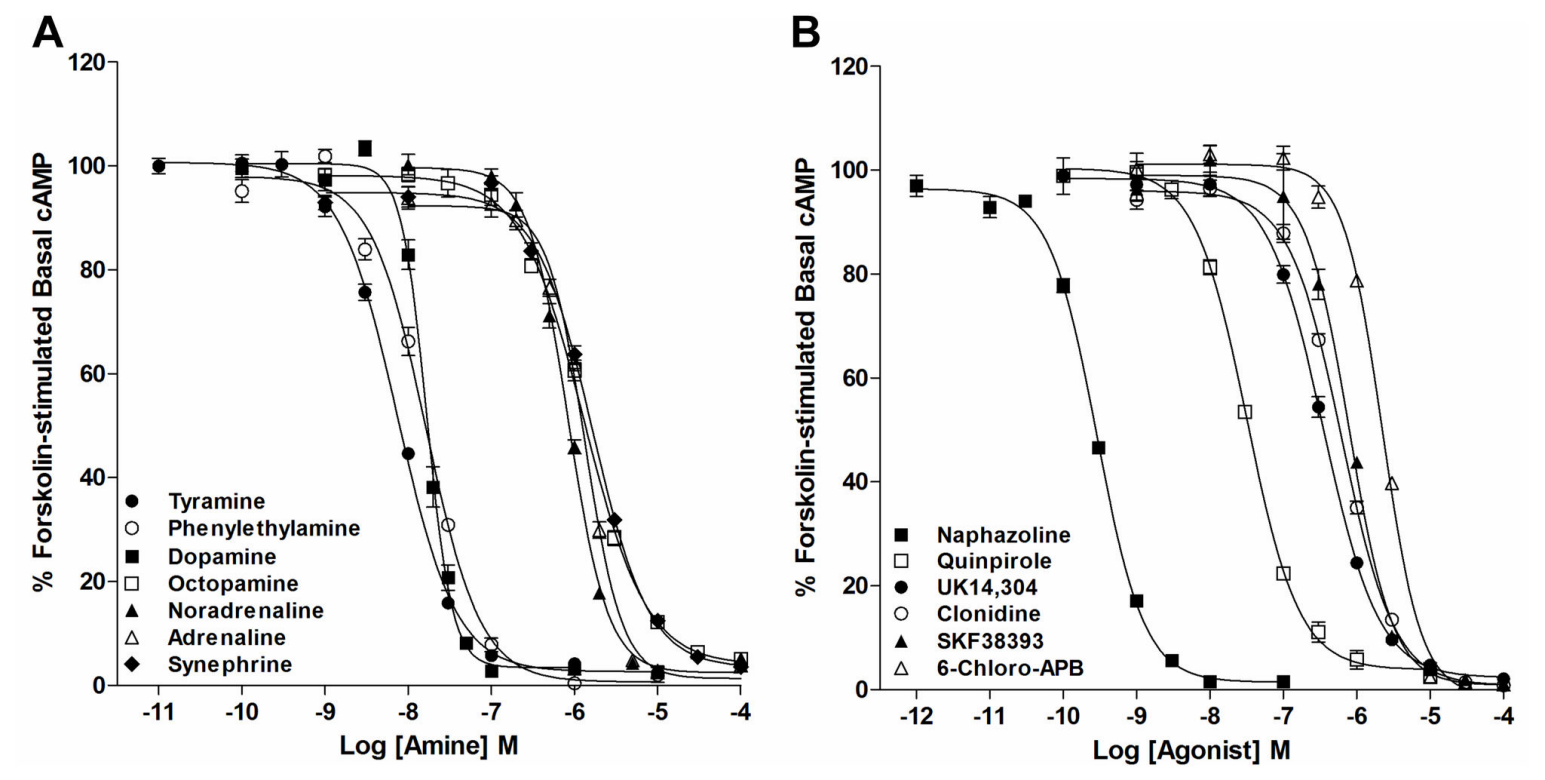
Effect of biogenic amines and synthetic agonists on forskolin-stimulated cAMP levels in AmphiAmR11-expressing CHO-K1 cells. Cells were pre-incubated with 100 µM IBMX for 20 min, followed by incubation with 10 µM forskolin and 100 µM IBMX in the presence of increasing concentrations of various biogenic amines (A) or synthetic agonists (B) for a further 20 min. Data are expressed as the mean ± SEM. *n* ≥ 3.

Since the AmphiAmR11 receptor shows structural similarities to vertebrate α_2_-adrenergic receptors [5], we examined its ability to regulate intracellular cyclic AMP levels when stably expressed in a CHO-K1 cell line and when activated by a wide range of naturally occurring biogenic amines. It can be seen that AmphiAmR11 coupled to a decrease in forskolin-stimulated cAMP levels upon stimulation with various biogenic amines (Figure 1A). Full concentration response curves showed that tyramine, phenylethylamine and dopamine with EC_50_’s of 7.47x10^-9^ M, 1.65x10^-8^ M and 1.67x10^-8^ M, respectively, were more potent than noradrenaline, octopamine, adrenaline and synephrine with EC_50_’s of 8.44x10^-7^ M, 1.30x10^-6^ M, 1.34x10^-6^ M and 1.65x10^-6^ M, respectively (Figure 1A). Histamine at 1 µM had no effect on forskolin-stimulated cAMP levels in AmphiAmR11-expressing CHO-K1 cells (Figure S1A in File S1). Control experiments showed that the biogenic amines used on AmphiAmR11-expressing CHO-K1 cells had no significant effect on forskolin-stimulated cAMP levels in wild type CHO-K1 cells [10]. 5-HT was not used in the assay since CHO-K1 cells have endogenous 5HT_1B_ receptors [26]. 

#### Synthetic agonist specificity

To investigate the pharmacological properties of AmphiAmR11, a range of synthetic agonists known to activate vertebrate adrenergic and dopaminergic receptors were screened for their ability to modulate forskolin-stimulated cAMP levels in AmphiAmR11-expressing CHO-K1 cells. The α-adrenergic agonist, naphazoline, and the D_2_-dopaminergic agonist, quinpirole, at 1 µM were found to be the most the most effective agonists (Figure S1B in File S1). Full concentration response curves showed that the rank order of potency (measured as EC_50_) was: naphazoline (2.87x10^-10^ M) >> quinpirole (3.17x10^-8^ M) > UK14,304 (3.50x10^-7^ M) = clonidine (6.23x10^-7^ M) = SKF38393 (7.92x10^-7^ M) > 6-Chloro-APB (2.23x10^-6^ M) (Figure 1B). Phenylephrine and isoproterenol at 1 µM were found to have little or no effect on forskolin-stimulated cAMP levels in AmphiAmR11-expressing CHO-K1 cells (Figure S1B in File S1). Control experiments showed that the synthetic agonists used in the present study had no significant effect on forskolin-stimulated cAMP levels in non-transfected wild type cells [10]. 

#### Synthetic antagonist specificity

**Figure 2:**
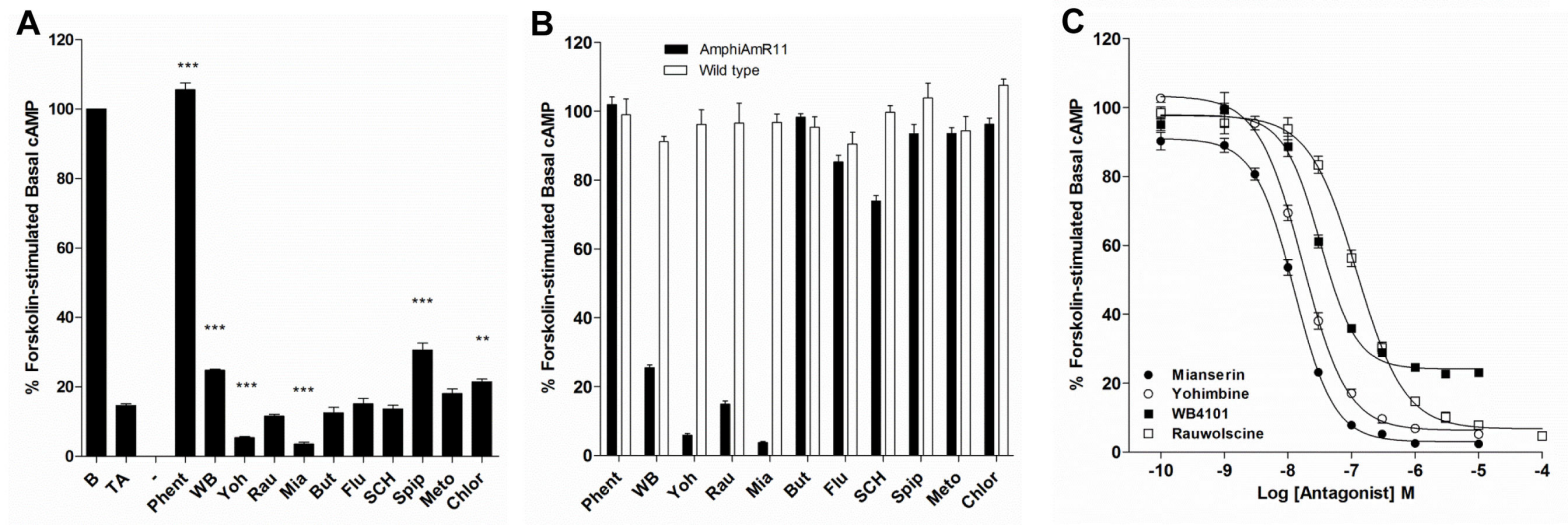
Effect of antagonists on forskolin-stimulated cAMP levels in AmphiAmR11-expressing CHO-K1 cells. (A) AmphiAmR11-expressing CHO-K1 cells were pre-incubated with 100 µM IBMX and 1 μM antagonist for 20 min, followed by incubation with 30 nM tyramine, 1 μM antagonist, 10 µM forskolin and 100 µM IBMX for a further 20 min. The basal value in the absence of agonist and antagonist is shown as 100% and the tyramine-only response in the absence of antagonist is shown for comparison. (B) AmphiAmR11-expressing (black bars) and wild type (open bars) CHO-K1 cells were pre-incubated with 100 µM IBMX and 1 μM antagonist for 20 min, followed by incubation with 1 μM antagonist, 10 µM forskolin and 100 µM IBMX for a further 20 min. (C) AmphiAmR11-expressing CHO-K1 cells were pre-incubated with 100 µM IBMX for 20 min, followed by incubation with 10 µM forskolin and 100 µM IBMX in the presence of increasing concentrations of antagonists for a further 20 min. Data are expressed as the mean ± SEM. (A) and (B) *n* > 5. (C) *n* = 3-4. **, *p* < 0.01; ***, *p* < 0.001. B, F-12 Hams-treated basal; TA, tyramine; Phent, phentolamine; WB, WB4101; Yoh, yohimbine; Rau, rauwolscine; Mia, mianserin; But, butaclamol; Flu, flupenthixol; SCH, SCH23390; Spip, spiperone; Meto, metoclopramide; Chlor, chlorpromazine.

Various classical adrenergic and dopaminergic antagonists were screened for their ability to block the tyramine-induced inhibition of forskolin-stimulated cAMP levels in AmphiAmR11-expressing CHO-K1 cells. The α-adrenergic antagonist, phentolamine, was found to fully block the tyramine-induced response, while WB4101, spiperone and chlorpromazine were found to have partial blocking effects (Figure 2A). The α-adrenergic antagonists, yohimbine, rauwolscine and mianserin, and the dopaminergic antagonists, butaclamol, flupenthixol, SCH23390 and metoclopramide were found to have no significant blocking effect at the receptor. However, yohimbine and mianserin appeared to enhance the tyramine-induced inhibition of forskolin-stimulated cAMP levels (Figure 2A) suggesting that the antagonists may have agonist properties at the receptor. To test this, the antagonists were screened for their ability to decrease forskolin-stimulated cAMP levels in the absence of agonist. It can be seen that WB4101, yohimbine, rauwolscine, mianserin and to a lesser extent, SCH23390, could inhibit forskolin-stimulated cAMP levels in AmphiAmR11-expressing CHO-K1 cells, while the other antagonists had no significant effect (Figure 2B, black bars). The effects of the antagonists were confirmed to be AmphiAmR11-specific since they had no significant effect on forskolin-stimulated cAMP levels in wild type CHO-K1 cells (Figure 2B, open bars). 

To confirm the agonist properties of WB4101, yohimbine, rauwolscine and mianserin at AmphiAmR11, full concentration-response curves were produced (Figure 2C). The rank order of potency (measured as EC_50_) was: mianserin (1.26x10^-8^ M) = yohimbine (1.71x10^-8^ M) > WB4101 (3.20x10^-8^ M) > rauwolscine (1.21x10^-7^ M). Mianserin, yohimbine and rauwolscine were found to act as full agonists at AmphiAmR11 since they inhibited forskolin-stimulated cAMP levels by greater than 90 %. In contrast, WB4101 only inhibited forskolin-stimulated cAMP levels by approximately 75 % suggesting WB4101 acted as a partial agonist at the receptor.

An attempt was made to determine if yohimbine (full agonist) and WB4101 (partial agonist) had any blocking effect at the receptor (Figure S2A, B in File S1). It was hoped that any blocking effect by the antagonists would be revealed if the concentration of the antagonist was reduced, to reduce its agonist effects. As the yohimbine concentration decreased, the agonist effects were reduced (Figure S2A in File S1, black bars). Decreasing the concentration of yohimbine also led to the tyramine-induced level of cAMP, when in the presence of yohimbine, to be reduced, but only to the level induced by tyramine alone (Fig, S2A in File S1, striped bars versus open bar). Thus, yohimbine appeared to have no blocking effect at the receptor. For WB4101, as its concentration was decreased, its agonist properties at the receptor were also reduced (Figure S2B in File S1, black bars). However, the reduction in WB4101 agonist properties did not reveal any further inhibitory effect on the tyramine-induced response. Instead, a decrease in WB4101 concentration led to an increase in the tyramine-induced effect, to the level of the tyramine-only response (Figure S2B in File S1, striped bars versus open bar). Thus, it appeared that WB4101 had a small blocking effect on the tyramine-induced reduction in cAMP levels in AmphiAmR11-expressing cells but only to the level of its own agonist activity. 

#### Effect of pertussis toxin on agonist activation

**Figure 3:**
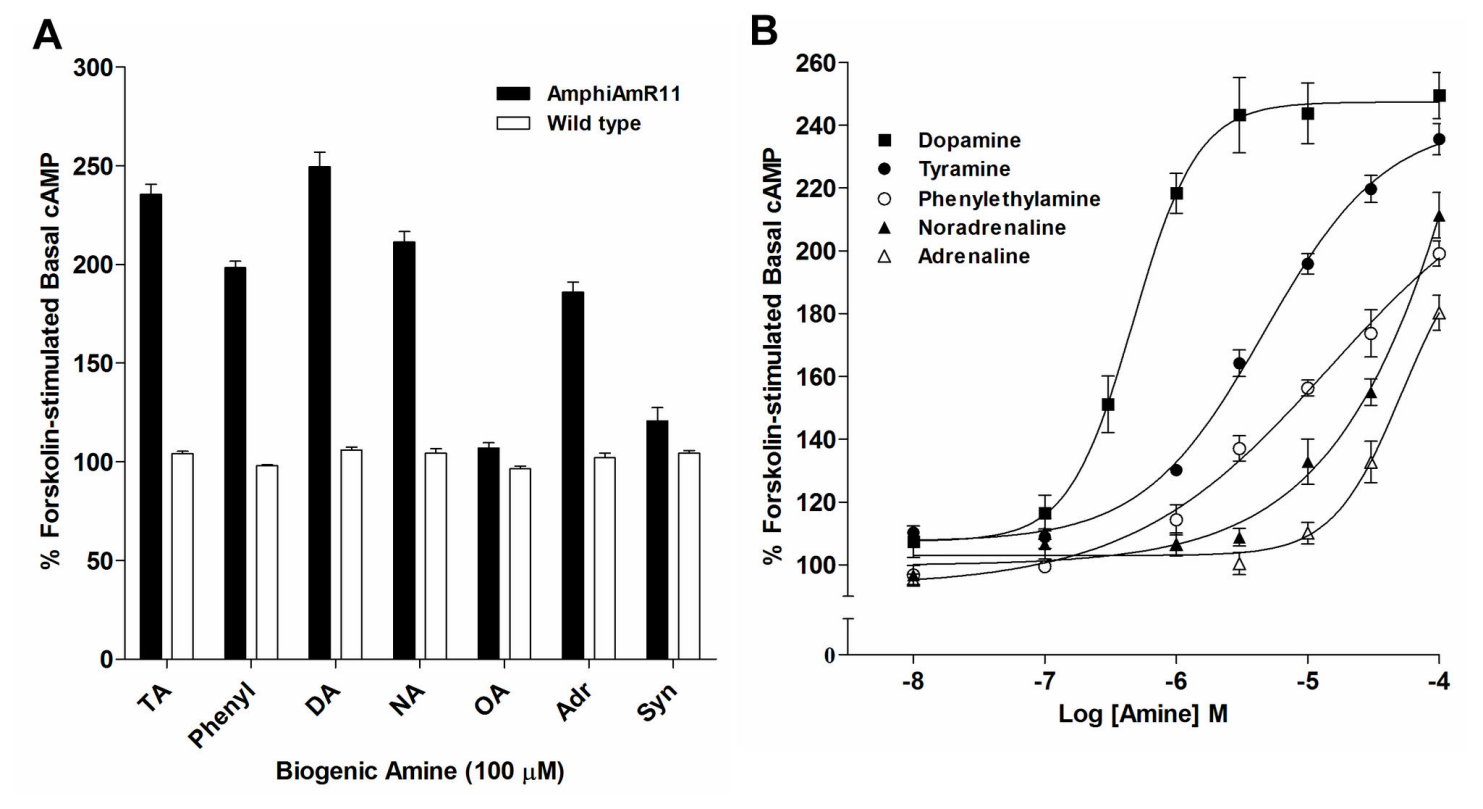
Effect of pertussis toxin on forskolin-stimulated cAMP levels in AmphiAmR11-expressing and wild type CHO-K1 cells. (A) AmphiAmR11-expressing (black bars) and wild type (open bars) CHO-K1 cells were pre-incubated with pertussis toxin (200 ng/mL) for 16 hours prior to incubation with 100 µM IBMX for 20 minutes. Cells were then stimulated with various biogenic amines at 100 µM and 10 µM forskolin, in the presence of 100 µM IBMX for 20 minutes. Data are expressed as the mean + SEM. (B) AmphiAmR11-expressing CHO-K1 cells were pre-incubated with pertussis toxin (200 ng/mL) for 16 h prior to incubation with 100 µM IBMX for 20 min. Cells were then stimulated with increasing concentrations of amines and 10 µM forskolin, in the presence of 100 µM IBMX for 20 min. Data are expressed as the mean ± SEM. n = 3-4. TA, tyramine; Phenyl, phenylethylamine; DA, dopamine; NA, noradrenaline; OA, octopamine; Adr, adrenaline; Syn, synephrine.

To determine whether AmphiAmR11-mediated inhibition of adenylyl cyclase activity was dependent on activation of G_i/o_ protein, AmphiAmR11-expressing CHO-K1 cells were pre-incubated with pertussis toxin, which inactivates G_i/o_ proteins [27]. Pertussis toxin treatment was found to inhibit the amine-mediated decreases in forskolin-stimulated cAMP levels described previously (Figure 3A). In addition, dopamine, tyramine, noradrenaline, phenylethylamine and adrenaline were found to couple AmphiAmR11 to an increase in forskolin-stimulated cAMP levels following pertussis toxin treatment (Figure 3A). In contrast, octopamine and synephrine at concentrations up to 100 µM did not induce an increase in forskolin-stimulated cAMP levels (Figure 3A), despite being as potent as noradrenaline and adrenaline in coupling AmphiAmR11 to adenylyl cyclase inhibition in the absence of pertussis toxin (Figure 1A). Concentration response curves showed that dopamine (EC_50_: 4.70x10^-7^ M) was the most potent amine at inducing the increase in forskolin-stimulated cAMP levels (Figure 3B). Tyramine, phenylethylamine, noradrenaline and adrenaline also showed concentration-dependent effects. However, even at concentrations of 100 µM, the phenylethylamine-, noradrenaline- and adrenaline-induced effect did not plateau, preventing potencies, or EC_50_’s, from being determined. Control experiments showed that the biogenic amines had no effect on forskolin-stimulated cAMP levels in wild type CHO-K1 cells following pertussis toxin treatment (Figure 3A). Thus, it appears that AmphiAmR11 may be able to switch its G protein coupling from G_i_ protein to G_s_ protein at high concentrations of some agonists. 

### Coupling of AmphiAmR11 to the activation of the mitogen-activated protein kinase (MAPK) pathway

#### Biogenic amine specificity

The AmphiAmR11 receptor was also assessed for its ability to activate the MAPK pathway. To determine the time course of MAPK activation, AmphiAmR11-expressing CHO-K1 cells were stimulated with 1 µM tyramine or 30 nM dopamine (maximal effect-producing concentrations, see below) for increasing lengths of time and the level of ERK1/2 phosphorylation induced was detected using Western blotting. An increase in ERK1/2 phosphorylation could be detected as early as 1 minute of agonist stimulation and peaked at approximately 5 minutes (Figure S3A, B, E in File S1). The level of ERK1/2 phosphorylation declined thereafter. By 30 minutes of tyramine stimulation, the level of ERK1/2 phosphorylation was approximately half that of its peak at 5 minutes. In contrast, dopamine displayed a more sustained ERK1/2 phosphorylation response. Since both dopamine and tyramine induced a peak ERK1/2 phosphorylation at approximately 5 minutes of stimulation, this time point was chosen for the remainder of the MAPK studies where agonist stimulation was involved.

**Figure 4:**
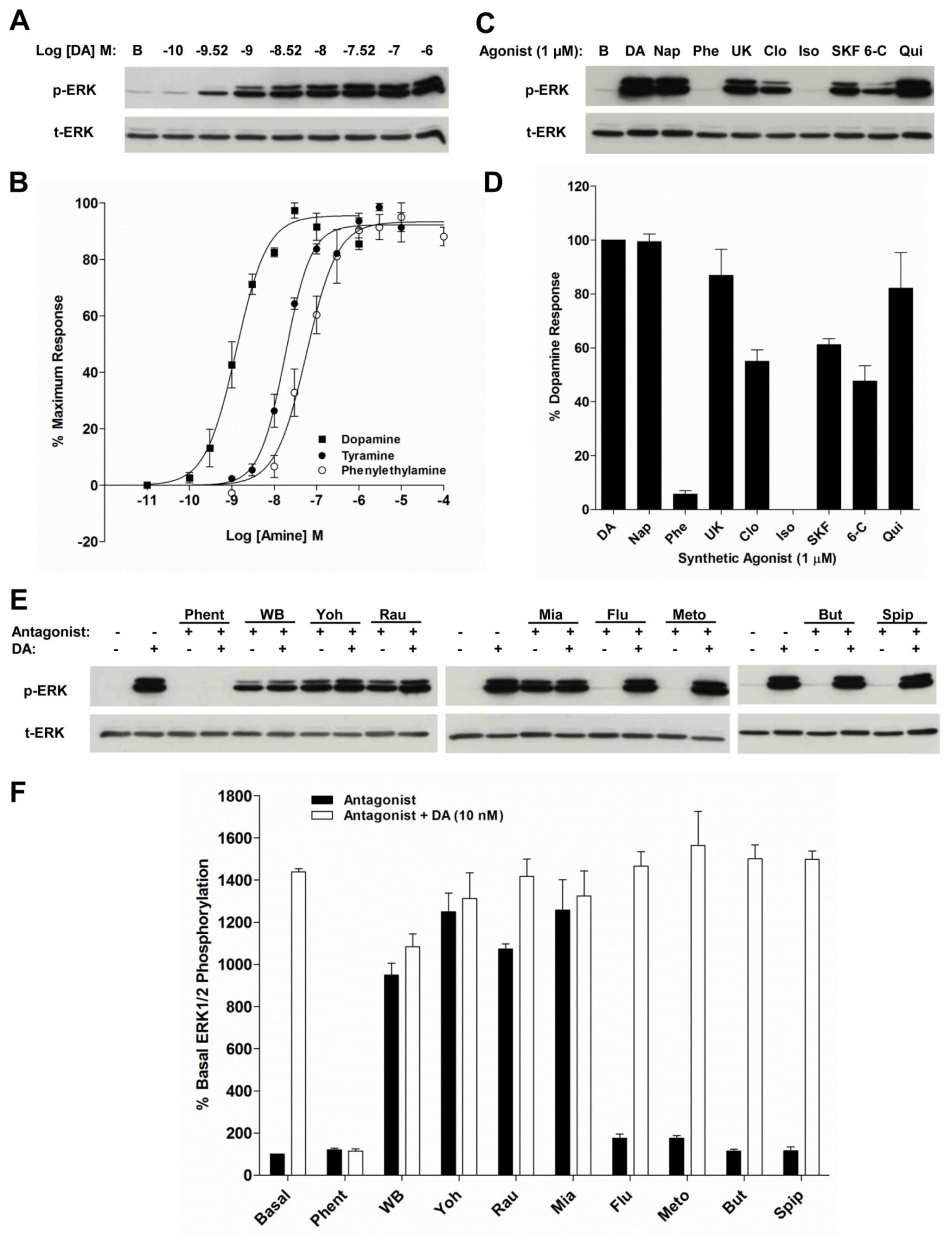
Effect of biogenic amines, synthetic agonists and antagonists on ERK1/2 activation in AmphiAmR11-expressing CHO-K1 cells. Cells were serum-starved for 2 h, prior to stimulation with various concentrations of biogenic amines (A and B) or 1 μM synthetic agonists (C and D) for 5 min. (E and F) Cells were serum-starved for 2 h, including incubation with 1 µM of antagonist or F-12 Hams control for the final 10 min. Cells were then stimulated with dopamine (10 nM) or F-12 Hams control in the presence of the antagonist for 5 min. Basal measurement in the absence of dopamine and antagonist (first black bar) and the dopamine-only response in the absence of antagonist (first open bar) are shown. Equal quantities of cell lysates were separated by SDS-PAGE and analysed for p-ERK or t-ERK by Western blotting. (A, C and E) Representative blots from three to four independent experiments. (B, D and F) Summaries of the quantified blots for each of the different experiments. Data are expressed as the mean ± SEM. n = 3-4. DA, Dopamine; Nap, naphazoline; Phe, phenylephrine; UK, UK14,304; Clo, clonidine; Iso, isoproterenol; SKF, SKF38393; 6-C, 6-Chloro-APB; Qui, quinpirole; Phent, phentolamine; WB, WB4101; Yoh, yohimbine; Rau, rauwolscine; Mia, mianserin; Flu, flupenthixol; Meto, metoclopramide; But, butaclamol; Spip, spiperone.

Concentration response curves for dopamine, tyramine and phenylethylamine showed that dopamine (EC_50_: 1.27x10^-9^ M) was more potent than tyramine and phenylethylamine (EC_50_: 1.82x10^-8^ M and 5.78x10^-8^ M, respectively) at inducing ERK1/2 phosphorylation in AmphiAmR11-expressing CHO-K1 cells (Figure 4A, B). The effect of noradrenaline, adrenaline, octopamine, synephrine and histamine on ERK1/2 phosphorylation in AmphiAmR11-expressing CHO-K1 cells was also assessed. At 10 nM, noradrenaline, adrenaline, octopamine, synephrine and histamine were not able to increase the level of ERK1/2 phosphorylation above that of the basal condition (Figure S4A, B in File S1). In control experiments, the amines were found to have no significant effect on ERK1/2 phosphorylation in wild type CHO-K1 cells [10]. Thus, dopamine is the most potent amine at coupling AmphiAmR11 to ERK1/2 phosphorylation. 

#### Synthetic agonist specificity

Classical adrenergic and dopaminergic synthetic agonists were screened for their ability to couple AmphiAmR11 to ERK1/2 phosphorylation. Dopamine was used as a reference compound in these studies since it was shown to be the most potent amine at coupling AmphiAmR11 to the MAPK pathway (Figure 4B). At 1 µM, naphazoline, quinpirole and UK14,304 were found to be the most effective of the synthetic agonists and induced ERK1/2 phosphorylation to a similar level to that induced by dopamine (Figure 4C, D). SKF38393, clonidine and 6-Chloro-APB also coupled AmphiAmR11 to ERK1/2 phosphorylation but were less effective than naphazoline, UK14,304 and quinpirole. Phenylephrine and isoproterenol had little or no effect on ERK1/2 activation. The synthetic agonists were also screened at 100 nM to determine their rank order of effectiveness. At 100 nM, only naphazoline increased the ERK1/2 phosphorylation level above that of the basal condition (Figure S4C, D in File S1). Thus, the rank order of effectiveness of the synthetic agonists to induce ERK1/2 phosphorylation was; naphazoline >> UK14,304 = quinpirole > SKF38393 = clonidine = 6-chloro-APB >> phenylephrine. 

#### Synthetic antagonist specificity

Adrenergic and dopaminergic antagonists were screened for their ability to block dopamine-induced ERK1/2 activation in AmphiAmR11-expressing CHO-K1 cells. Phentolamine was the only antagonist found to fully block the dopamine-induced ERK1/2 response while WB4101 was found to have a partial effect (Figure 4E, F). In contrast, yohimbine, rauwolscine, mianserin, flupenthixol, metoclopramide, butaclamol and spiperone had no significant effect on the dopamine-induced response. Thus, the results obtained parallel those of the cAMP assay described previously. 

The antagonists were also screened for their effect on ERK1/2 phosphorylation in the absence of any agonist since four of the antagonists, WB4101, yohimbine, rauwolscine and mianserin, were found to have agonist properties in coupling AmphiAmR11 to adenylyl cyclase inhibition (Figure 2). In parallel with the cAMP assay, the same four antagonists at 1 μM coupled AmphiAmR11 to an increase in ERK1/2 phosphorylation in the absence of dopamine (Figure 4E, F, black bars). Yohimbine and mianserin were the most effective ligands to induce ERK1/2 phosphorylation, followed by rauwolscine and WB4101. Phentolamine, flupenthixol, metoclopramide, butaclamol and spiperone did not exhibit any agonistic properties on ERK1/2 phosphorylation. Thus, the properties of the antagonists in the MAPK assay were consistent with their properties in the cAMP assay. The antagonists had no significant effect on ERK1/2 phosphorylation in wild type CHO-K1 cells [10]. 

#### The effects of MAPK pathway inhibitors

**Figure 5:**
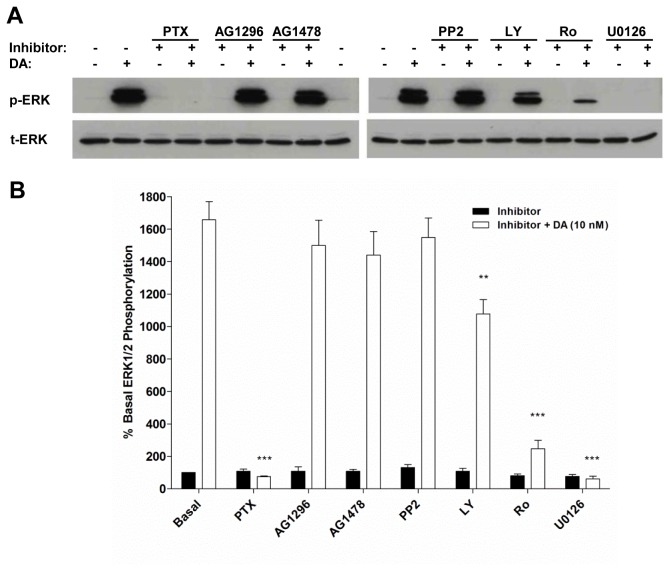
Effect of pertussis toxin and signalling inhibitors on dopamine-induced ERK1/2 activation in AmphiAmR11-expressing CHO-K1 cells. For the pertussis toxin experiment, AmphiAmR11-expressing CHO-K1 cells were pre-incubated with pertussis toxin (200 ng/ml) for 16 hours prior to serum-starvation for 2 hours in the absence of the toxin. Cells were then stimulated for 5 mins with 10 nM dopamine (open bar) or F-12 Hams control (black bar). For all other inhibitors, AmphiAmR11-expressing CHO-K1 cells were serum starved for 2 hours including incubation with 10 µM inhibitor or F-12 Hams control for the final 20 (AG1296 and AG1478) or 30 (PP2, LY294002, Ro31-8220, U0126, U73122 and U73343) minutes. Cells were then stimulated for 5 minutes with 10 nM dopamine (black bars) or F-12 Hams control (open bars) in the presence of the inhibitor. Basal measurement in the absence of dopamine and inhibitor (first black bar) and the dopamine-only response in the absence of inhibitor (first open bar) are shown. (A) Representative blots from three or four independent experiments. (B) Summary of the quantified blots. **, *p* < 0.01 versus dopamine-only; ***, *p* <0.001 versus dopamine-only. Data are expressed as the mean + SEM. n = 3 - 4. DA, dopamine; PTX, pertussis toxin; Ro, Ro 31-8220; LY, LY294002.

Various inhibitors of key signalling molecules were used to determine the pathway involved in dopamine-induced ERK1/2 activation in AmphiAmR11-expressing CHO-K1 cells. Pre-incubation of AmphiAmR11-expressing CHO-K1 cells with pertussis toxin abolished the ERK1/2 response induced at 5 minutes of dopamine stimulation (Figure 5A, B). Pertussis toxin pre-treatment also abolished tyramine- and dopamine-induced ERK1/2 phosphorylation at multiple time points (Figure S3C, D, E in File S1). Thus, G_i/o_ protein activation is required for AmphiAmR11-mediated ERK1/2 phosphorylation following stimulation with 10 nM and 30 nM dopamine, and 1 µM tyramine. 

Tyrphostin inhibitors, AG1296 and AG1478, which inhibit platelet-derived growth factor (PDGF) and epidermal growth factor (EGF) receptors, respectively, were used to determine if AmphiAmR11 couples to the MAPK pathway via transactivation of PDGF and EGF receptors. Since neither inhibitor had a significant effect on dopamine-induced ERK1/2 phosphorylation (Figure 5), AmphiAmR11-mediated ERK1/2 phosphorylation appeared not to be dependent on the transactivation of PDGF or EGF receptors. Similarly, the Src tyrosine kinase inhibitor, PP2, had no effect on dopamine-mediated ERK1/2 activation (Figure 5), suggesting Src activation was not required for AmphiAmR11-mediated ERK1/2 phosphorylation.

To identify whether AmphiAmR11 couples to ERK1/2 activation via activation of phosphatidylinositol-3-kinase (PI3K) and/or protein kinase C (PKC), AmphiAmR11-expressing CHO-K1 cells were pre-incubated with LY294002 or Ro 31-8220, which inhibit PI3K and PKC, respectively. LY294002 treatment significantly reduced the level of dopamine-induced ERK1/2 phosphorylation (Figure 5). Ro 31-8220 had a greater effect than LY294002 and reduced the level of dopamine-induced ERK1/2 phosphorylation to near basal levels (Figure 5). Similarly, the MEK1/2 inhibitor, U0126, abolished the dopamine-induced ERK1/2 response (Figure 5). Thus, AmphiAmR11 couples to ERK1/2 phosphorylation via activation of pathways involving PKC, PI3K and MEK1/2.

### Coupling of AmphiAmR11 to calcium mobilisation

#### Biogenic amine specificity

**Figure 6:**
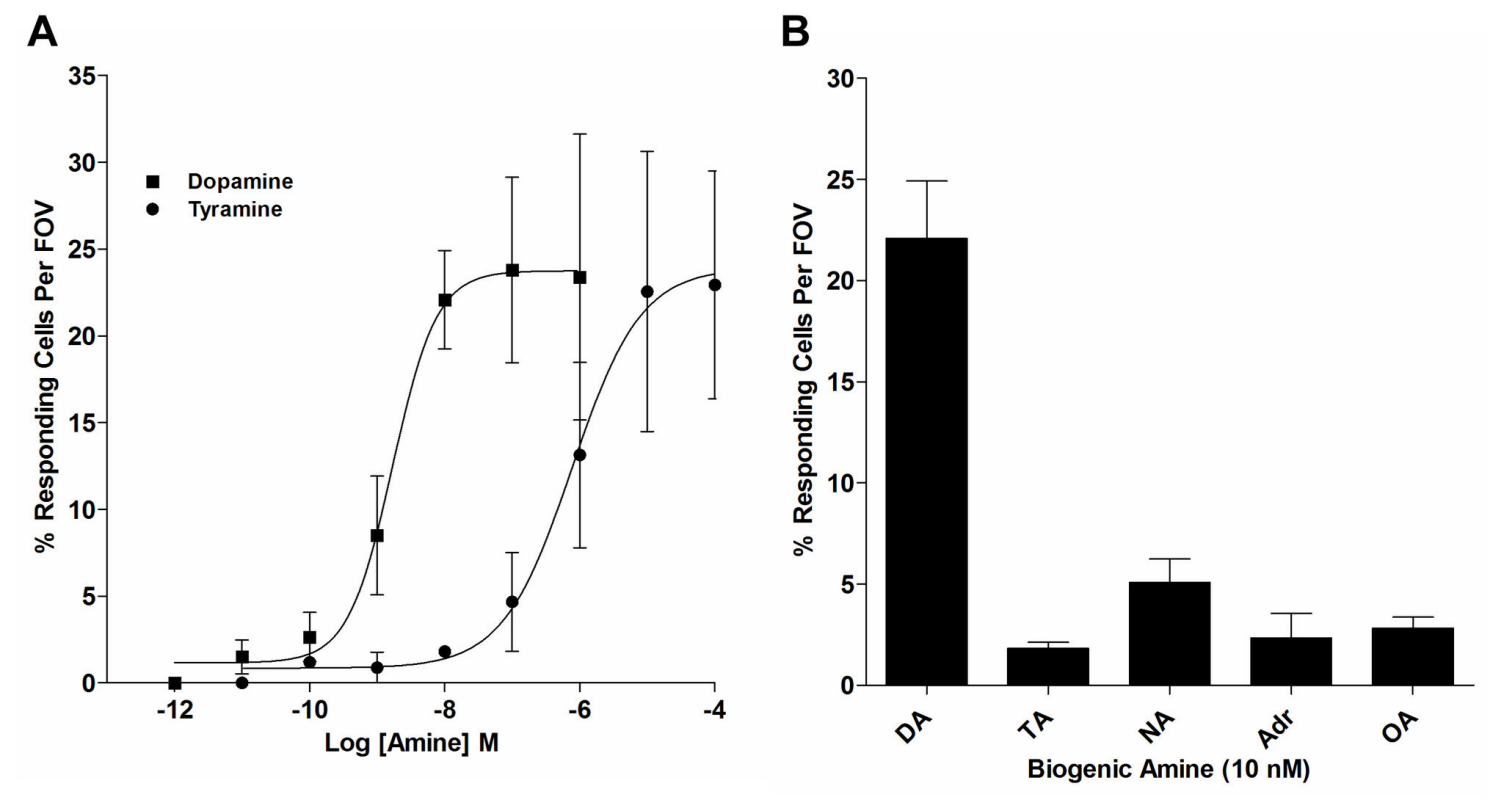
Effect of biogenic amines and calcium pathway inhibitors on calcium mobilisation in AmphiAmR11-expressing CHO-K1 cells. AmphiAmR11-expressing cells were exposed to increasing concentrations (A) or 10 nM (B) of biogenic amines for 3 minutes and the number of responding cells per field of view were calculated. Data are expressed as the mean ± SEM. *n* ≥ 3. FOV, field of view; DA, dopamine; TA, tyramine; NA, noradrenaline; Adr, adrenaline; OA, octopamine.

While AmphiAmR11 shows structural similarities to α_2_-ARs [5], the high potency of dopamine in coupling AmphiAmR11 to the cAMP and MAPK pathways suggests the receptor is functionally more similar to D_2_-dopamine receptors. Since vertebrate D_2_-dopamine receptors have been shown to mediate changes in intracellular calcium levels [17], AmphiAmR11 was also assessed for its ability to modulate calcium levels in CHO-K1 cells. While both dopamine and tyramine were found to couple AmphiAmR11 to calcium mobilisation, the responses were found to vary between individual cells and not all of the cells in a field of view responded (Figure S5A in File S1). This made it difficult to measure the parameters that are usually measured when assaying changes in intracellular calcium levels, such as peak height, the onset of the calcium response and the area under the curve. To overcome this and to enable the most potent amine at coupling AmphiAmR11 to calcium mobilisation to be identified, the number of cells that responded per field of view was measured (28,29). It can be seen that dopamine and tyramine induced concentration-dependent increases in the percentage of responding cells per field of view (Figure 6A). Dopamine with an EC_50_ of 1.68x10^-9^ M was found to be approximately 450-fold more potent than tyramine (EC_50_: 7.67x10^-7^ M). Noradrenaline, adrenaline and octopamine were also assessed for their ability to couple AmphiAmR11 to calcium mobilisation. At 10 nM, noradrenaline, adrenaline and octopamine were less effective than dopamine at inducing a calcium response (Figure 6B). Control experiments showed that the biogenic amines at 10 µM had no effect on intracellular calcium levels in wild type CHO-K1 cells (Figure S5B in File S1) and that all AmphiAmR11 expressing cells showed a high level of receptor in the plasma membrane with smaller amounts in the cytoplasm (Figure S6 in File S1). Thus, dopamine is the most potent amine at coupling AmphiAmR11 to calcium mobilisation in CHO-K1 cells.

## Discussion

The present study describes the characterisation of the amphioxus GPCR, AmphiAmR11, expressed in CHO-K1 cells. AmphiAmR11 was found to couple to a decrease in forskolin-stimulated cAMP levels, a characteristic of vertebrate and invertebrate D_2_-like dopamine receptors [30-34] and also invertebrate Oct/Tyr receptors [35-38]. Tyramine, phenylethylamine and dopamine, were found to be the most potent biogenic amines. AmphiAmR11 was also found to couple to ERK1/2 activation and calcium mobilisation, and in both pathways, dopamine was found to be the most potent biogenic amine. This is similar to vertebrate D_2_-dopamine receptors, which have also been shown to mediate increases in ERK1/2 phosphorylation and calcium mobilisation upon dopamine stimulation [17]. However, in the present study we cannot rule out the possibility that the changes in intracellular calcium could be a secondary effect to changes in the MAPKinase pathway. Thus, AmphiAmR11 appears functionally most similar to vertebrate D_2_-dopamine receptors [17], despite showing highest structural similarity to vertebrate α_2_-ARs [5].

While dopamine and tyramine displayed similar potencies in the cAMP assay, dopamine was 10-fold and 450-fold more potent than tyramine in the MAPK assay and calcium assay, respectively. This variation in potencies could be indicative of a relatively recent phenomenon of GPCR signalling, known as ‘agonist-specific coupling’ or ‘functional selectivity’, amongst many other terms [37,39,40]. Functional selectivity refers to the ability of different ligands to preferentially couple the same receptor to different pathways. For example, octopamine is more potent than tyramine at coupling the Drosophila Oct/Tyr receptor to calcium mobilisation but tyramine is more potent than octopamine at coupling the receptor to adenylyl cyclase inhibition [37]. It is thought that different ligands stabilise a different conformation of the receptor which then leads to the enhancement of one signalling pathway over another [39,40]. Hence, dopamine and tyramine could be stabilising different conformations of AmphiAmR11, resulting in different pathways being preferentially activated by the two amines. 

The α-AR agonist, naphazoline was found to be the most potent agonist in both the cAMP and MAPK assays. Since AmphiAmR11 shows high sequence similarity to α_2_-ARs, it is perhaps not unexpected that an α-AR agonist can activate it. In addition, naphazoline was found to be the most potent agonist at activating the closely related amphioxus receptor, AmphiAmR4 [10]. The D_2_-dopamine receptor agonist, quinpirole, was also an effective agonist in the AmphiAmR11-activated cAMP pathway. The potency obtained for quinpirole-induced decrease in forskolin-stimulated cAMP levels is consistent with its affinity at mammalian D_2_-dopamine receptors [41]. In contrast, quinpirole was found not to activate the D_2_-like dopamine receptor from Drosophila, DD2R [32]. This may suggest that AmphiAmR11 is more similar to mammalian D_2_-dopamine receptors than invertebrate D_2_-like receptors. 

The α-AR antagonist, phentolamine, was the only antagonist found to fully block tyramine-mediated decreases in forskolin-stimulated cAMP levels and dopamine-mediated increases in ERK1/2 phosphorylation levels. In parallel to the effects of naphazoline mentioned above, it is not unexpected that an α-AR antagonist can bind and have a blocking effect at AmphiAmR11 since AmphiAmR11 is similar in sequence to α_2_-ARs. For the dopaminergic antagonists used, AmphiAmR11 displayed properties similar to invertebrate D_2_-like receptors. For example, spiperone is a potent antagonist at mammalian D_2_ receptors [17,41] but has little or no effect on dopamine-mediated signalling via the Drosophila DD2R receptor [32] or via the *C. elegans* CeDOP2S and CeDOP2L receptors [34]. In the present study, spiperone had only a small blocking effect on tyramine-induced decreases in forskolin-stimulated cAMP levels and no effect on dopamine-induced ERK1/2 activation. In addition, the dopaminergic antagonists, SCH23390 and chlorpromazine, had no significant effect on AmphiAmR11-expressing cells and were also found not to affect DD2R-, CeDOP2S- and CeDOP2L-mediated signalling [32,34]. Furthermore, the non-selective dopamine receptor antagonists, butaclamol and flupenthixol, had only a partial effect at the invertebrate D_2_-like receptors when used at a high concentration (10 µM) [32,34] and no effect on AmphiAmR11-mediated signalling. 

The α-AR antagonist, WB4101, and α_2_-AR antagonists, yohimbine, rauwolscine and mianserin, displayed unique properties at AmphiAmR11; they had little or no blocking effect at the receptor but instead, acted as agonists. Concentration response curves showed that the four supposed antagonists displayed similar potency values to those of the most potent biogenic amines, dopamine and tyramine. Although to a lesser degree than that seen for AmphiAmR11, previous studies have shown that ligands classified as antagonists at one type of receptor can have agonist properties at other receptors. For example, the α-AR antagonist, phentolamine, was shown to have agonist properties at the amphioxus receptor, AmphiAmR4 [10], and the Drosophila receptors OctβRs, CG6989, CG6919 and CG7078 [21]. In addition, rauwolscine and yohimbine were shown to have partial agonist activity at a human 5HT_1A_ receptor [42].

Similar to D_2_-dopamine receptors (43), AmphiAmR11 was found to couple to adenylyl cyclase inhibition and ERK1/2 phosphorylation via activation of Gi protein. The use of pertussis toxin also revealed that AmphiAmR11 could mediate increases in cAMP levels in CHO-K1 cells. Dopamine was the most potent amine at inducing this response and was found to couple AmphiAmR11 to an increase in forskolin-stimulated cAMP levels at concentrations considered physiological (less than 1 µM). Tyramine, phenylethylamine, noradrenaline and adrenaline could also couple AmphiAmR11 to an increase in cAMP levels but only at concentrations above 1 µM. These results suggest that AmphiAmR11 may be able to switch its coupling from G_i/o_ proteins to G_s_ proteins at high concentrations of some agonists. Other G_i_-coupled receptors, including α_2_-ARs [44], D_3_-dopamine receptors [45] and the closely related AmphiAmR4 receptor [10], have also been shown to switch their coupling to G_s_ proteins at high agonist concentrations. 

Inhibitors of various signalling molecules potentially involved in the activation of ERK1/2 in AmphiAmR11-expressing CHO-K1 cells were used to determine the pathway downstream of G_i_ protein activation. The PI3K inhibitor, LY294002, partially reduced dopamine-induced ERK1/2 activation in AmphiAmR11-expressing CHO-K1 cells, suggesting that PI3K activation was required for a maximal ERK1/2 response. This parallels the pathway induced by vertebrate D_2_- and D_3_-dopamine receptors, since LY294002 and an unrelated PI3K inhibitor, wortmannin, partially reduce D_2_- and D_3_-mediated ERK1/2 activation in CHO cells [46,47]. Cussac et al. [46] also showed that D_3_-dopamine receptor-mediated ERK1/2 activation in CHO cells was dependent on PKC activation but independent of Src activation. This parallels the findings in the present study since AmphiAmR11-mediated ERK1/2 activation was reduced to near basal levels in the presence of the PKC inhibitor, Ro 31-8220, but was not affected by the Src inhibitor, PP2. AmphiAmR11-mediated ERK1/2 activation was also found to be dependent on the activation of MEK1/2, since the MEK1/2 inhibitor, U0126, abolished the dopamine-induced ERK1/2 response. The similarity between the vertebrate D_3_-dopamine receptor- and the AmphiAmR11-induced ERK1/2 pathways provides support for AmphiAmR11 being similar to the vertebrate D_2_ class of dopamine receptors. 

AmphiAmR11 is the first D_2_-like dopamine receptor to be characterised from amphioxus. Its ability to bind, and be activated by, dopamine is likely to be physiologically relevant since amphioxus has three populations of dopaminergic neurons in its central nervous system, and expresses a relatively high level of dopamine throughout its body [7]. In addition, previous studies have shown that amphioxus has several D_1_-type dopamine receptors, including two vertebrate-type D_1_-dopamine receptors [8,9]. Tyramine, the metabolic precursor of octopamine, was also found to activate AmphiAmR11 at physiologically relevant concentrations (less than 1 µM). While it is unknown if tyramine is synthesized in amphioxus, the high levels of octopamine [7] suggest that it is likely to be. Thus, if the potential expression of tyramine has a functional role in amphioxus and it is synthesized in an area where AmphiAmR11 is localised, it is possible that tyramine could also act at the receptor *in vivo*. However, further studies on the cellular localization of tyramine and its release from specific neurons are required before a physiological role for tyramine can be established in the amphioxus nervous system.

The characterisation of a D_2_-like dopamine receptor from amphioxus contrasts with the suggestion that D_2_-dopamine receptors have been secondarily lost in the protochordate lineage [48]. In fact, it is possible that more than one D_2_-like receptor exists in amphioxus since Nordstrom et al. [6] identified a further two sequences that clustered with vertebrate D_2_- and D_3_-dopamine receptors in a phylogenetic tree. Interestingly, AmphiAmR11 was found not to cluster with the two D_2_/D_3_-like amphioxus receptors identified by Nordstrom et al. [6] nor was AmphiAmR11 found to cluster with vertebrate D_2_/D_3_ receptors in the phylogenetic tree generated by Burman et al. [5]. Instead, AmphiAmR11 was found to cluster with four other amphioxus receptors, AmphiAmR4, -5, -6 and -9, off the main branch of a phylogenetic tree leading to vertebrate α_2_-ARs [5]. One of the receptors from this group, AmphiAmR4, has already been characterised and shows highest functional similarity to α_2_-ARs [10]. We believe that the present study provides insight into the evolution of α_2_-ARs and supports the hypothesis that α_2_-ARs evolved from D_2_-dopamine receptors [12,13]. The high sequence similarity of AmphiAmR11 to α_2_-ARs but its functional similarity to D_2_-dopamine receptors, shown by the high potency of dopamine in each of the pathways assayed, may suggest that the receptor represents a transition state between D_2_-dopamine and α_2_-adrenergic receptors.

## Supporting Information

File S1 **Supplementary Information Bayliss and Evans containing Figures S1-S6.** Figure S1. Effect of biogenic amines (A) and synthetic agonists (B) on forskolin-stimulated cAMP levels in AmphiAmR11-expressing CHO-K1 cells. Figure S2. Effect of yohimbine and WB4101 on tyramine-induced decreases in forskolin-stimulated cAMP levels in AmphiAmR11-expressing CHO-K1 cells. Figure S3. Time courses for the effect of dopamine and tyramine on ERK1/2 phosphorylation in AmphiAmR11-expressing CHO-K1 cells. Figure S4. The relative effectiveness of the biogenic amines and synthetic agonists on ERK1/2 activation in AmphiAmR11-expressing CHO-K1 cells. Figure S5. Calcium mobilisation in AmphiAmR11-expressing and wild type CHO-K1 cells. Figure S6. Localization of AmphiAmR11 in CHO-K1 cells.
(DOCX)
