# Supplementary material for: Characterisation of AmphiAmR11, an Amphioxus (Branchiostoma floridae) D2-Dopamine-Like G Protein-Coupled Receptor
Source: PLoS One. 2013 Nov 12;8(11):e80833. doi: 10.1371/journal.pone.0080833 (PMC3827198; doi:10.1371/journal.pone.0080833)
Supplement: File S1 — Supplementary Information Bayliss and Evans containing Figures S1-S6. Figure S1. Effect of biogenic amines (A) and synthetic agonists (B) on forskolin-stimulated cAMP levels in AmphiAmR11-expressing CHO-K1 cells. Figure S2. Effect of yohimbine and WB4101 on tyramine-induced decreases in forskolin-stimulated cAMP levels in AmphiAmR11-expressing CHO-K1 cells. Figure S3. Time courses for the effect of dopamine and tyramine on ERK1/2 phosphorylation in AmphiAmR11-expressing CHO-K1 cells. Figure S4. The relative effectiveness of the biogenic amines and synthetic agonists on ERK1/2 activation in AmphiAmR11-expressing CHO-K1 cells. Figure S5. Calcium mobilisation in AmphiAmR11-expressing and wild type CHO-K1 cells. Figure S6. Localization of AmphiAmR11 in CHO-K1 cells. (DOCX) [file pone.0080833.s001.docx]

**File S1: Supporting Information on line PLOS ONE**

Characterisation of AmphiAmR11, an amphioxus (*Branchiostoma floridae*) D_2_-dopamine-like G protein-coupled receptor

By

Asha L. Bayliss and Peter D. Evans

Peter.evans@babraham.ac.uk

**Supplementary Figures S1-S6**

**Supplementary Figure Legends Bayliss and Evans**

**Figure S1.** **Effect of biogenic amines (A) and synthetic agonists (B) on forskolin-stimulated cAMP levels in AmphiAmR11-expressing CHO-K1 cells.** Cells were pre-incubated with 100 µM IBMX for 20 min, followed by incubation with 10 µM forskolin and 100 µM IBMX in the presence of 1 µM biogenic amines (A) or synthetic agonists (B) for a further 20 min. Data are expressed as the mean + SEM. *n* ≥ 3.

**Figure S2. Effect of yohimbine and WB4101 on tyramine-induced decreases in forskolin-stimulated cAMP levels in AmphiAmR11-expressing CHO-K1 cells**. Cells were pre-incubated with 100 µM IBMX in the presence or absence of varying concentrations of the antagonists, yohimbine (A) or WB4101 (B), for 20 minutes, followed by incubation with either varying concentrations of antagonist (black bars) or 30 nM tyramine plus varying concentrations of antagonist (striped bars) in the presence of 10 µM forskolin and 100 µM IBMX for a further 20 minutes. The basal value in the absence of agonist and antagonist (grey bar) and the tyramine-only value (open bar) are shown. Data are expressed as the mean + SEM. *n* = 3. Yoh, yohimbine; TA, tyramine; WB, WB4101.

**Figure S3. Time courses for the effect of dopamine and tyramine on ERK1/2 phosphorylation in AmphiAmR11-expressing CHO-K1 cells.** (A and B) AmphiAmR11-expressing CHO-K1 cells were serum-starved for 2 hours, prior to stimulation with tyramine or dopamine at the specified concentration for the specified length of time. Equal quantities of cell lysates were separated by SDS-PAGE and analysed for pERK or tERK by Western blotting. (C and D): AmphiAmR11-expressing CHO-K1 cells were incubated with pertussis toxin (200 ng/mL) for 16 hours followed by stimulation as described for Figures A and B. (A to D) Representative blot for each condition from three to four independent experiments. (E) Summary of the quantified blots. Data are expressed as the mean ± SEM.  *n* = 3-4. PTX, pertussis toxin; Min, minutes.

**Figure S4.** **The relative effectiveness of the biogenic amines and synthetic agonists on ERK1/2 activation in AmphiAmR11-expressing CHO-K1 cells.** Cells were serum-starved for 2 h, prior to stimulation with 10 nM biogenic amines (A and B) or 100 nM synthetic agonists (C and D) for 5 min. Equal quantities of cell lysates were separated by SDS-PAGE and analysed for p-ERK or t-ERK by Western blotting. (A and C) Representative blots from three to four independent experiments. (Band D) Summary of the quantified blots. Data are expressed as the mean + SEM. *n =* 3-4. DA, Dopamine; TA, tyramine; Phen, Phenylethylamine, NA, noradrenaline; Adr, adrenaline; OA, octopamine; Syn, synephrine; His, histamine; Nap, naphazoline; Phe, phenylephrine; UK, UK14,304; Clo, clonidine; Iso, isoproterenol; SKF, SKF38393; 6-C, 6-Chloro-APB; Qui, quinpirole.

**Figure S5. Calcium mobilisation in AmphiAmR11-expressing and wild type CHO-K1 cells.** (A) Representative traces for agonist-induced calcium mobilisation in AmphiAmR11-expressing cells. During agonist stimulation, some cells in a field of view coupled to calcium mobilisation (represented by Cells 1-3), while others did not (represented by Cell 4). (B) Wild type CHO-K1 cells were stimulated with various biogenic amines at 10 µM for 8 minutes followed by stimulation with adenosine trisphosphate (ATP) at 10 µM for 5 minutes. *n* ≥ 3.

**Figure S6. Localization of AmphiAmR11 in CHO-K1 cells.** AmphiAmR11-expressing CHO-K1 cells were fixed with 4% paraformaldehyde and stained with an anti-V5-FITC antibody (green). Nuclei were stained with DAPI (blue). All cells from the clonal cell line used in the present study showed strong staining in the periphery of the cells consistent with its function as a biogenic amine receptor since biogenic amines are cell membrane impermeable. Additional staining was observed particularly in the perinuclear region of the cells. Wild type cells showed no staining.

**Bayliss and Evans Figure S1**

**A**

**B**

**Bayliss and Evans Figure S2**

**B**

**A**

**Bayliss and Evans Figure S3**

**A**

**C**

**Tyramine 1 µM + PTX**

**Time (Min): 0 1 2 5 10 15 20 30**

**Tyramine 1 µM**

**Time (Min): 0 1 2 5 10 15 20 30**


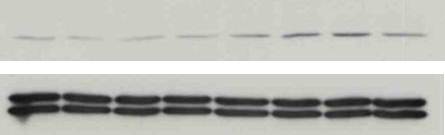

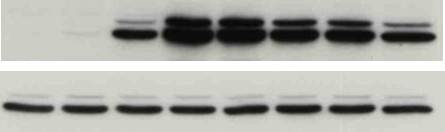


**p-ERK**

**t-ERK**

**p-ERK**

**t-ERK**

**D**

**B**

**Dopamine 30 nM + PTX**

**Dopamine 30 nM**

**t-ERK**

**p-ERK**

**Time (Min): 0 1 2 5 10 15 20 30**

**t-ERK**

**p-ERK**

**Time (Min): 0 1 2 5 10 15 20 30**


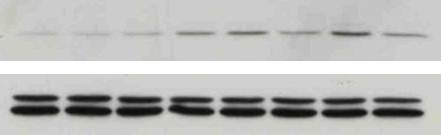

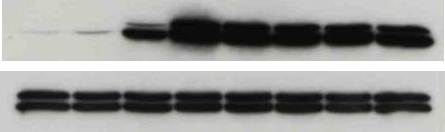


**E**

**Bayliss and Evans Figure S4**

**C**

**A**

**
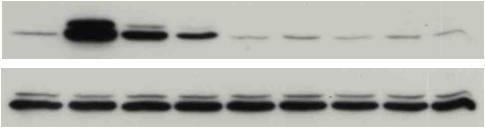

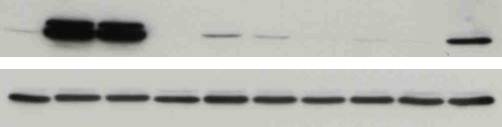
**

**Agonist: B DA Nap Phe UK Clo Iso SKF 6-C Qui**

**Amine: B DA TA Phen NA Adr OA Syn His**

**t-ERK**

**p-ERK**

**p-ERK**

**t-ERK**

**D**

**B**

**Bayliss and Evans Figure S5**

**A**

**B**

**Bayliss and Evans Figure S6**

**
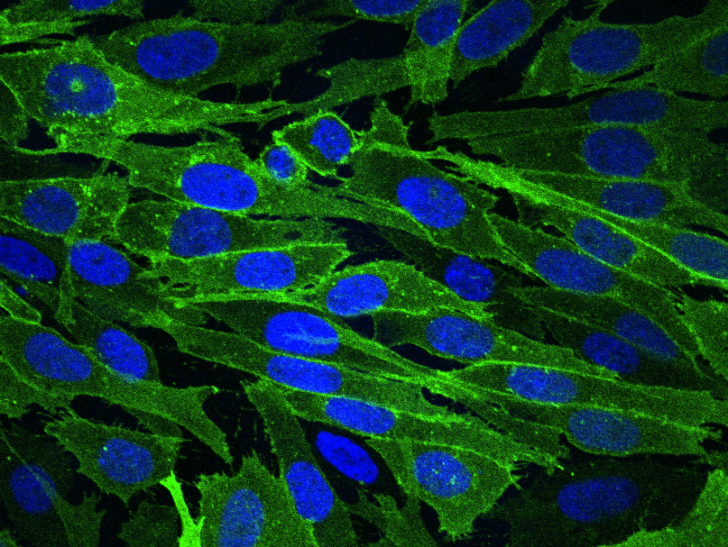
**
